# Pulmonary fat embolism and related effects during femoral intramedullary surgery: An experimental study in dogs

**DOI:** 10.3892/etm.2013.1143

**Published:** 2013-06-05

**Authors:** FEIHU ZHOU, JUN JI, QING SONG, ZHIYONG PENG, GUOQIANG ZHANG, YAN WANG

**Affiliations:** 1 Department of Critical Care Medicine, Chinese People’s Liberation Army General Hospital, Beijing 100853;; 2 Department of Anesthesiology, Air Force General Hospital, Beijing 100142, P.R. China;; 3 The Clinical Research, Investigation, and Systems Modeling of Acute Illness (CRISMA) Center, Department of Critical Care Medicine, University of Pittsburgh, Pittsburgh, PA, USA;; 4 Department of Orthopaedics, Chinese People’s Liberation Army General Hospital, Beijing 100853, P.R. China

**Keywords:** pulmonary fat embolism, hemodynamics, cytokine, extravascular lung water

## Abstract

The aim of the present study was to develop an animal model of pulmonary fat embolism (PFE) caused by femoral intramedullary procedures, and to investigate the initial changes in the hemodynamics, cytokines and risk factors of PFE. Sixteen dogs were randomly divided into two groups: Group A (intramedullary reaming and bone cement injection, n=8) and Group B (surgical approach without opening the medullary cavity, n=8). The hemodynamics, arterial blood gases and relevant cytokines were evaluated, and the lungs were examined using Oil Red O staining. In the animals of Group A, the heart rate, central venous pressure, mean pulmonary arterial pressure, pulmonary capillary wedge pressure and extravascular lung water (EVLW) were increased compared with the baseline levels, while the mean arterial pressure was decreased immediately following the reaming and bone cement infusion (P<0.05). Furthermore, there was a significant reduction in the pH and the arterial oxygen tension (PaO_2_), and a significant increase in the arterial carbon dioxide tension (PaCO_2_; P<0.05 for all) following the bilateral intramedullary surgery. The EVLW was correlated with the PaO_2_ (P<0.001) and PaCO_2_ (P=0.046). Following surgery, there was a significant increase in tumor necrosis factor-α (TNF-α), interleukin-1β (IL-1β) and IL-6 levels in Group A (P<0.05). However, there were no significant changes in these parameters in Group B. The parameters tested, with the exception of pH, were significantly different in Group A compared with those in Group B (P<0.05) following the bilateral intramedullary surgery. Oil Red O staining was positive for all animals in Group A and negative for those in Group B. Femoral intramedullary surgery may induce PFE and subsequently affect hemodynamics and arterial blood gases. EVLW was correlated with the PaO_2_ (P<0.001) and the PaCO_2_ (P=0.046). These results demonstrated that EVLW and cytokines may serve as predictors of the development of fat embolism syndrome (FES).

## Introduction

Fat embolism syndrome (FES) is a severe complication of orthopedic surgery or trauma. It occurs in ∼1.29% of patients with multiple fractures, particularly those with a femoral fracture (1). Typically, it triggers the development of pulmonary fat embolism (PFE), which has been reported to be present in 82% of trauma patients (2). The acute consequences of fat embolism are hemodynamic disorders and, in severe cases, right-sided heart failure or hypoxemia (3,4). Intraoperative cardiovascular deterioration, as a result of the pulmonary embolization of bone marrow fat, is a potential complication (5). Although numerous animal models that reveal many aspects of PFE have been developed (5–9), the underlying mechanisms of the syndrome, including the early hemodynamic effects, the potential inflammatory responses and the risk factors of PFE, are not fully understood. The aim of the present study was to develop a clinically relevant animal model, and to investigate the initial changes in the hemodynamics, cytokines, arterial blood gases and the risk factors involved in PFE that are caused by femoral intramedullary procedures.

## Subjects and methods

### Subjects

The study was approved by the Animal Research Committee of the Chinese People’s Liberation Army (PLA) General Hospital (Beijing, China). Sixteen healthy male outbred dogs (weight, 14.7–22.3 kg), were randomly divided into two groups: Group A (intramedullary reaming and bone cement injection, n=8) and Group B (surgical approach without opening the medullar cavity, n=8).

### Surgical procedure and measurement of biochemical parameters

**Figure 1.**
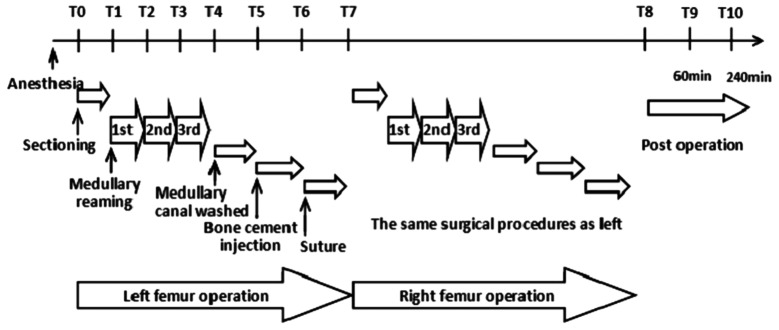
Surgical approaches and associated time points. T0, prior to surgery; T1, prior to unilateral medullary reaming; T2, following the first unilateral medullary reaming; T3, following the second unilateral medullary reaming; T4, following the third unilateral medullary reaming; T5, prior to unilateral bone cement injection; T6, 5 min following unilateral bone cement injection; T7, following unilateral femur surgery; T8, following bilateral femur surgery; T9, 60 min following bilateral femur surgery; T10, 240 min following bilateral femur surgery.

Anesthesia was induced with an intravenous injection of sodium pentobarbital (30 mg/kg), and maintained with a continuous infusion (5 mg/kg/min). Each dog was orally intubated with an endotracheal tube, and mechanically ventilated with a Servo-i Ventilator [Maquet, Inc., Rastatt, Germany; basic setting: volume control ventilation, initial rate 18/min and tidal volume 12 ml/kg; I:E=1:2; positive end expiratory pressure (PEEP), 4 cm H_2_O; and FiO_2_, 40%] during the experiment. A pulmonary artery catheter (Arrow International, Inc., Reading, PA, USA) was inserted through the external jugular vein. A pulse contour cardiac output (PiCCO) hot dilated catheter was inserted via femoral cut-down and connected to the PiCCO monitor (PiCCO Plus, Pulsion Medical System AG, Munich, Germany) for the determination of pulmonary and systemic hemodynamics. The biochemical parameters, including hemodynamic parameters, arterial blood gases and cytokine levels, were measured. Hemodynamic data were recorded at specific time points, and blood samples were collected for the measurement of arterial blood gases and cytokine levels at such time points (Fig. 1). The biochemical parameters comprised heart rate (HR), mean arterial pressure (MAP), central venous pressure (CVP), mean pulmonary arterial pressure (MPAP), pulmonary capillary wedge pressure (PCWP) and extravascular lung water (EVLW). In the arterial blood gas tests, the pH, arterial oxygen tension (PaO_2_) and arterial carbon dioxide tension (PaCO_2_) were evaluated. In addition, the cytokine levels that were measured were those of tumor necrosis factor-α (TNF-α), interleukin-1β (IL-1β) and IL-6. The time points at which measurements were taken were as follows: T0, prior to surgery; T1, prior to unilateral medullary reaming; T2, following the first unilateral medullary reaming; T3, following the second unilateral medullary reaming; T4, following the third unilateral medullary reaming; T5, prior to unilateral bone cement injection; T6, 5 min following unilateral bone cement injection; T7, following unilateral femur surgery; T8, following bilateral femur surgery; T9, 60 min following bilateral femur surgery; T10, 240 min following bilateral femur surgery.

In Group A, the greater lateral trochanter of the left femur was resected through a muscle gap by the posterior lateral femur approach. The intertrochanteric fossa and entry of the medullary canal were exposed and followed by intramedullary reaming to one-half the length of the femur. The medullary canal was washed with saline to remove destroyed myeloid tissues, and dried with gauze. Bone cement (Tianjin Synthetic Material Research Institute, Hexi, China) was prepared by manually stirring the powder into the solution at a ratio of 2:1, until a dough was formed, which was then used to fill the medullary canal. When completely solidified, the incision was sutured. The same surgical approaches were repeated in the right femurs of the dogs in Group A. The femurs of the dogs in Group B were subjected to the same surgical methods as those in Group A, with the exception of the resection of the greater trochanter and the opening of the medullary cavity. The anesthetized dogs were sacrificed by the intravenous injection of 5 ml potassium chloride (15 meq) 240 min following bilateral femur surgery. Necropsies were performed immediately following sacrifice, to obtain the right and left lungs. Oil Red O staining was used to identify the presence of fatty deposits in these tissues.

### Statistical analysis

Data are presented as the mean ± standard deviation. The mean differences between the study groups and within groups among different time points were analyzed by analysis of variance (ANOVA) for repeated measures. Correlations between variables were analyzed and expressed as correlation coefficients. P<0.05 was considered to indicate a statistically significant difference. The statistical analysis was performed using SPSS 13.0 for Windows (SPSS, Inc., Chicago, IL, USA).

## Results

**Figure 2.**
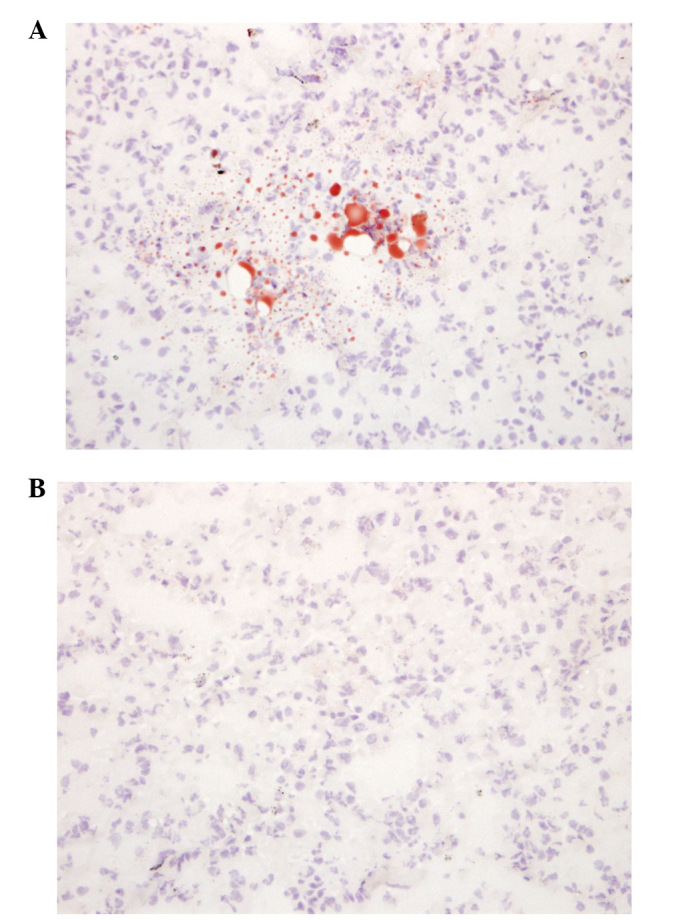
Lung sections of animals from (A) Group A and (B) Group B. (A) Shows multiple fat emboli stained by Oil Red O. (B) No staining indicated. Original magnification, ×40.

All animals survived the experimental procedure. In all lung sections from Group A, fat emboli stained by Oil Red O were observed, which demonstrated that the animals had developed PFE (Fig. 2A). However, no fat emboli were identified in any of the lung sections from Group B (Fig. 2B).

**Figure 3.**
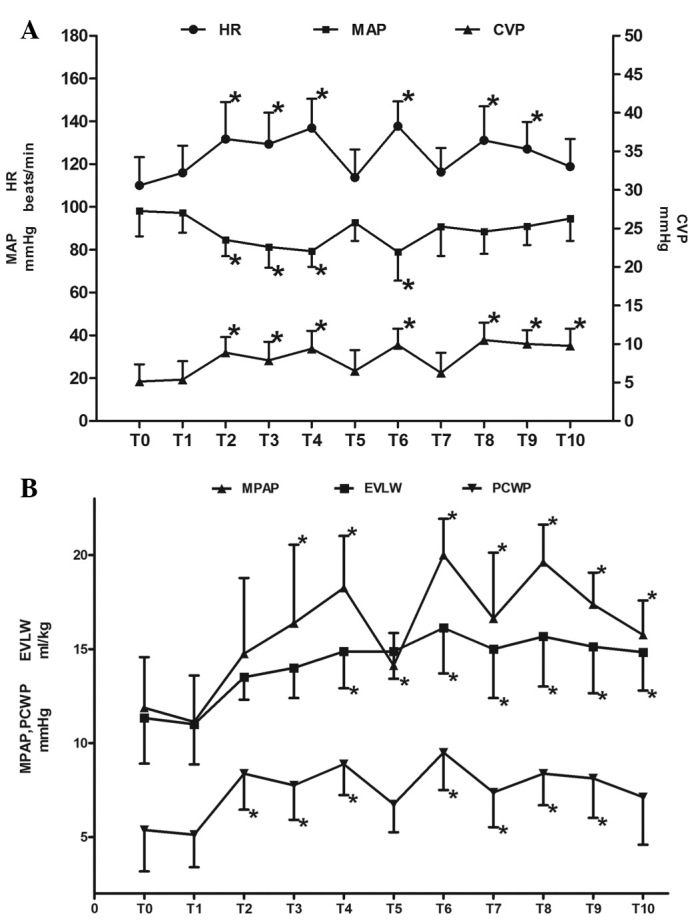
Alterations in the hemodynamic paramters at various time points during and following intramedullary surgery in Group A. (A) Changes in heart rate (HR), mean arterial pressure (MAP) and central venous pressure (CVP). (B) Changes in mean pulmonary arterial pressure (MPAP), extravascular lung water (EVLW) and pulmonary capillary wedge pressure (PCWP). ^*^P<0.05 vs. T0. T0, prior to surgery; T1, prior to unilateral medullary reaming; T2, following the first unilateral medullary reaming; T3, following the second unilateral medullary reaming; T4, following the third unilateral medullary reaming; T5, prior to unilateral bone cement injection; T6, 5 min following unilateral bone cement injection; T7, following unilateral femur surgery; T8, following bilateral femur surgery; T9, 60 min following bilateral femur surgery; T10, 240 min following bilateral femur surgery.

Figs. 3A and B demonstrate the changes in the hemodynamics in Group A. The HR, CVP, MPAP, PCWP and EVLW increased, while the MAP decreased, immediately following the reaming and bone cement infusion, compared with those values at T0. In addition, the values at T4 and T6 were significantly different from those at T0 (P<0.05). Changes in the HR, MAP and PCWP were observed at T10; the values of the parameters were no longer significantly different from those at T0 (P>0.05). By contrast, the CVP, MPAP and EVLW remained high at this time point, compared with those at T0 (P<0.05). However, there were no significant differences in the hemodynamics in Group B (P>0.05). Significant differences were identified in the HR, MAP, and PCWP at T8, as well as in the CVP, MPAP and EVLW at T8, T9 and T10 in Group A (Table I), compared with those in Group B.

Group A demonstrated reductions in the pH and the PaO_2_, but an increase in the PaCO_2_, at T8, which were significantly different compared with those at T0 (P<0.05; Table II). Although there was an increase in the pH and the PaO_2_, and a reduction in the PaCO_2_, at T10, the PaCO_2_ (46.6±7.5 mmHg) remained high and was significantly different compared with that at T0 (P<0.05). The PaO_2_ was correlated with the EVLW [linear correlation coefficient, −0.5664; 95% confidence interval (CI), −12.181 to −3.614; P<0.001]. Similar results were identified between PaCO_2_ and EVLW (linear correlation coefficient, 0.3560; 95% CI, 0.02409–2.216; P=0.046). However, no significant changes were observed in the arterial blood gases in Group B. Significant differences were identified in the PaCO_2_ at T8, as well as in the PaO_2_ at T7 and T8, between Group B and Group A (Table II).

**Figure 4.**
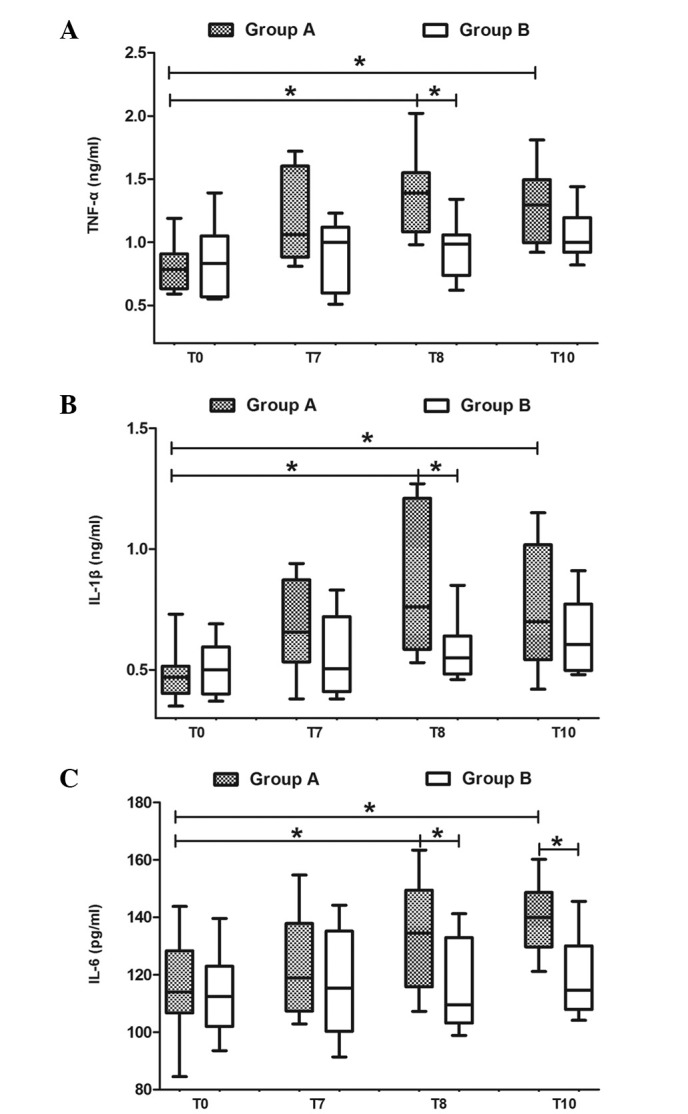
Alterations in the cytokine levels at various time points during and following intramedullary surgery in Groups A and B. Changes in the plasma concentration of (A) tumor necrosis factor-α (TNF-α), (B) interleukin-1β (IL-1β) and (C) IL-6. ^*^P<0.05 between groups A and B. T0, prior to surgery; T7, following unilateral femur surgery; T8, following bilateral femur surgery; T10, 240 min following bilateral femur surgery.

Following the surgery, there were increases in the TNF-α, IL-1β and IL-6 levels in Group A. Significant differences were observed in the TNF-α levels at T8 and T10, as well as in the IL-1β and IL-6 levels at T8 and T10, compared with those at T0 (P<0.05; Fig. 4). However, there were no significant differences in the levels of these cytokines in Group B. There were significant differences in the TNF-α and IL-1β levels at T8, as well as in the IL-6 levels at T8 and T10, in Group A, when compared with those in Group B (P<0.05; Fig. 4).

## Discussion

The present study demonstrated that femoral intramedullary surgery may induce PFE and subsequently affect hemodynamics and arterial blood gases. The EVLW was correlated with the PaO_2_ and the PaCO_2_, and may serve as a predictor of the development of FES. Cytokines were significant during these procedures, and may be further predictors for the development of FES. To the best of our knowledge, this study is the first to demonstrate a correlation between the EVLW, cytokines and FES, providing potential methods for the diagnosis and treatment of FES.

The clinical manifestation of PFE ranges from no impairment, in mild cases, to death, in severe cases (4). As for the pathogenesis of fat embolism, there are currently two hypotheses; the mechanical occlusion theory and the biochemical theory (10). In the most severe case, extensive embolization has been shown to be associated with lung injury and acute respiratory distress syndrome (ARDS) (11). With an enhanced understanding of the disease, an increasing number of studies are currently investigating the inflammatory response in PFE (12–15). In a study on fat embolism in rats, Liu *et al* demonstrated that serum TNF-α, IL-1β and neutrophil elastase levels were increased in pulmonary alveolus irrigating solution, in accordance with an altered lung weight and pulmonary hypertension, and an increased capillary filtration coefficient (14). Blankstein *et al* identified that the combination of hemorrhagic shock, resuscitation and fat embolism elicited neutrophil activation, infiltration of the alveoli by polymorphonuclear leukocytes and inflammatory cytokine expression, in bronchoalveolar lavage fluid (12). Furthermore, serum IL-6 is considered to be a potential early marker of fat embolism (13). The present study identified that serum TNF-α, IL-1β and IL-6 levels were higher than those at the baseline, following the surgical process. These results were consistent with other animal studies (13,14), as well as with certain clinical investigations (16,17). This suggests that these inflammatory cytokines are important during PFE, and subsequently lead to lung injury. Therefore, the early removal of these inflammatory mediators is a potential target of therapy.

Intraoperative cardiovascular deterioration, as a result of pulmonary embolization of bone marrow fat, is a potentially fatal complication during total hip and knee arthroplasty, intramedullary nailing and spine surgery (18). It is important to correct the hemodynamic changes caused by fat embolism. Therefore, comprehensive studies of hemodynamics and early interventions are required. Aebli *et al* conducted a study in which bone cement was injected into the L1 vertebral bodies of six sheep, and demonstrated that following the injection of the bone cement, there were reflective reductions in the HR and arterial pressure, and that the second reduction in the arterial pressure was caused by secondary PFE (19). Similar results were observed in the present study. Significant increases in the HR, CVP, MPAP, PCWP and EVLW and a significant reduction in the MAP occurred immediately following the reaming and bone cement infusion processes. It was thought that the changes were associated with the intramedullary procedures, leading to the release of bone marrow particles and the formation of microthrombi in the pulmonary artery. This was confirmed by Oil Red O staining of pulmonary sections from the current study animals. Wheelwright *et al* also concluded that pulmonary fat and marrow embolism were key factors of cardiorespiratory and hemodynamic instability, following canal pressurization (20). It is likely that the methylmethacry-late monomer, acting as a systemic vasodilator, is also a factor during this procedure (21).

Pathological examinations are able to reveal the presence of alveolar edema and hemorrhage with multiple fat droplet depositions and fibrin thrombi; however, chest radiography is unable to locate these during the early stages of fat embolism (16). EVLW is a useful measure of the accumulation of parenchymal lung edema, and there has previously been shown to be a significant correlation between EVLW measurements indexed to all body weights and the severity of lung injury in patients with ARDS (22). In the present study, the EVLW levels remained continuously high from the completion of the intramedullary procedure to 240 min following the bilateral femur surgery. Furthermore, following the bilateral femoral intramedullary procedure, there was a reduction in the pH and the PaO_2_, and an increase in the PaCO_2_. The cytokine levels, specifically those of TNF-α, IL-1β and IL-6, were also higher compared with those prior to surgery. Thus, the intramedullary surgery resulted in the formation of PFE and the release of inflammatory cytokines. This increased the permeability of the pulmonary microcirculation and the EVLW, which subsequently resulted in a reduction in the PaO_2_ and an increase in the PaCO_2_. The PaO_2_ and the PaCO_2_ were correlated with the EVLW (P<0.05), indicating that EVLW, as an independent index, may be valuable in the evaluation of pulmonary edema during the early stages of PFE.

The present study employed tracheal intubation, general anesthesia and controlled mechanical ventilation procedures. The basic settings of the ventilator remained unchanged, and therefore minimized the influence of other factors on respiration. The pathological results indicated that intramedullary procedures were able to evoke PFE, which mainly resulted in a reduced PaO_2_ and an increased PaCO_2_ during surgery. Similar results have been identified in other studies (6,23). A potential reason for the induction of PFE may be that a fat embolus entered the lung, which decreased the blood perfusion and exchange area, and subsequently increased the dead space area. Alternatively, in this study, as the basic settings of the ventilator remained unchanged, the pulmonary edema caused by fat embolism and the release of inflammatory cytokines may have resulted in an increased airway pressure and a decreased tidal volume. Bilateral procedures carry higher risks compared with unilateral ones.

Although the results of the present study indicated that the pulmonary tissue sections of all animals in Group A exhibited fat particles, the present embolism model did not lead to severe changes in clinical symptoms. Arterial blood gases and certain hemodynamic parameters, such as HR and MAP, improved 240 min following the bilateral procedures (T10). These results were in accordance with the fact that the majority of intramedullary reaming processes do not induce FES (24). Discordance between the clinical and experimental diagnosis of fat embolism, or ARDS, was mainly due to pulmonary infiltration (25,26) or compensation of the patient and the size of the fat embolus (27). In a clinical study, Kim revealed that 65% of patients who underwent bilateral knee joint replacement and 46% of patients who underwent unilateral knee joint replacement demonstrated positive Oil Red O staining of fat particles in their blood (28). Pitto *et al* studied patients with a femoral neck fracture who received cemented total hip joint replacement and identified, using transesophageal echocardiography (TEE), that 95% of the patients had fat embolism presentations (29). The aforementioned studies suggest a high prevalence of PFE induced by intramedullary procedures, with PFE as a potential complication of such procedures. The treatment options, such as clofibrate, dextran-40, ethyl alcohol, heparin and aspirin, have been evaluated previously and shown to cause no significant changes in the clinical outcomes of PFE and FES (27,30). Strict monitoring of the hemodynamics during surgery is essential if a patient has high risk factors, such as multiple injuries and infection, in the clinical setting. In the case of continuously abnormal hemodynamics, an early intervention is required to prevent fat embolism from developing into FES.

There were several limitations to this study. In Group B, the surgery was conducted without opening the medullary cavity. Therefore, it was not possible to compare the hemodynamic parameters of the two groups during the surgical procedure in the medullary cavity, such as medullary reaming and bone cement injection. However, comparisons between Groups A and B demonstrated that the animals in Group A were effective models in which to evaluate PFE through postmortem pulmonary tissues. Furthermore, the model allowed for the creation of fat embolism and the tracking of the changes in cytokine levels and arterial blood gas parameters during the surgical procedure. Another limitation of this study was that the dogs were kept alive for only four hours following surgery, and the risk factors were determined during the early stages of PFE. Therefore, it was not possible to draw conclusions regarding the development of fat embolism and inflammation over a period of several days following the procedure. The focus was on the initial impact of PFE, during or following the surgery. Therefore, further experimental studies in animals are required to investigate these possible events.

In conclusion, femoral intramedullary surgery may induce PFE and affect the hemodynamics and arterial blood gases, for example, by reducing the PaO_2_ and increasing the PaCO_2_. The EVLW was demonstrated to correlate with the PaO_2_ and the PaCO_2_, and may serve as a predictor for the development of FES. Positive Oil Red O staining in pulmonary tissue sections was observed in animals that received intramedullary surgery, indicating the high prevalence of PFE induced by such a surgical procedure. The serum concentrations of TNF-α, IL-1β and IL-6 increased following the intramedullary surgery, and these cytokines may therefore be a risk factor for the development of FES.

## Figures and Tables

**Table I.** Hemodynamic data summary for Groups A and B.

Parameter	Group	T0	T1	T7	T8	T9	T10
HR (beats/min)	A	110.13±13.18	116.00±12.71	116.38±11.17	131.13±15.91	127.13±12.68	118.88±12.84
	B	115.63±12.93	117.13±10.30	118.38±9.10	116.13±7.86^a^	120.62±10.41	117.50±10.18
MAP (mmHg)	A	98.13±11.79	97.25±9.32	90.88±13.71	88.50±10.41	91.00±8.72	94.63±10.42
	B	100.13±9.33	97.75±9.22	98.00±6.01	101.25±6.34^a^	99.38±6.25	101.38±5.73
CVP (mmHg)	A	5.13±2.23	5.38±2.39	6.25±2.60	10.50±2.27	10.00±1.77	9.75±2.25
	B	5.12±1.36	5.00±1.76	4.88±1.13	5.25±1.28^a^	5.50±1.51^a^	5.38±1.19^a^
MPAP (mmHg)	A	11.88±2.70	11.13±2.47	16.63±3.50	19.63±1.99	17.38±1.69	15.75±1.83
	B	10.88±2.90	10.63±1.85	11.13±2.99	12.12±2.18^a^	11.37±2.50^a^	11.87±3.04^a^
PCWP (mmHg)	A	5.38±2.20	5.13±1.73	7.38±1.85	8.37±1.69	8.13±2.10	7.12±2.53
	B	5.50±2.45	5.63±2.20	5.38±1.19	5.75±1.04^a^	6.13±1.36	6.63±1.41
EVLW (ml/kg)	A	11.33±2.42	11.00±2.14	15.00±2.61	15.67±2.66	15.13±2.47	14.83±2.04
	B	11.13±3.48	10.50±1.77	11.25±2.66^a^	10.75±2.49^a^	10.63±1.99^a^	11.37±1.77^a^

a P<0.05 indicates a significant difference between Groups A and B. T0, prior to surgery; T1, prior to unilateral medullary reaming; T7, following unilateral femur surgery; T8, following bilateral femur surgery; T9, 60 min following bilateral femur surgery; T10, 240 min following bilateral femur surgery. HR, heart rate; MAP, mean arterial pressure; CVP, central venous pressure; MPAP, mean pulmonary arterial pressure; PCWP, pulmonary capillary wedge pressure; EWLW, extravascular lung water.

**Table II.** Parameters of arterial blood gases analyzed at different time points in Groups A and B.

Parameter	Group	T0	T7	T8	T10
pH	A	7.420±0.075	7.370±0.062	7.336±0.072^a^	7.360±0.065
	B	7.399±0.068	7.414±0.066	7.386±0.088	7.396±0.047
PaO_2_ (mmHg)	A	191.1±34.1	180.1±38.7^a^	156.4±30.9^a^	186.5±34.2
	B	206.8±45.2	212.1±50.8^b^	208.3±49.1^b^	202.4±28.6
PaCO_2_ (mmHg)	A	36.6±6.0	43.6±7.7	48.4±7.6^a^	46.6±7.5^a^
	B	38.5±6.3	40.1±7.0	39.8±4.4^b^	39.3±2.4

a P<0.05 compared with the value at T0, and

b P<0.05 compared with the value in Group A. T0, prior to surgery; T7, following unilateral femur surgery; T8, following bilateral femur surgery; T10, 240 min following bilateral femur surgery. PaO_2_, arterial oxygen tension; PaCO_2_, arterial carbon dioxide tension.
